# Predictive value of readiness, importance, and confidence in ability to change drinking and smoking

**DOI:** 10.1186/1471-2458-12-708

**Published:** 2012-08-29

**Authors:** Nicolas Bertholet, Jacques Gaume, Mohamed Faouzi, Gerhard Gmel, Jean-Bernard Daeppen

**Affiliations:** 1 Department of Community Medicine and Health, Alcohol treatment center, Lausanne University Hospital, Beaumont 21b, P2, 02, Lausanne, 1011, Switzerland; 2 Center for Alcohol and Addiction Studies, Brown University, Providence, RI, USA

**Keywords:** Readiness to change, Importance of changing, Confidence in ability to change, Unhealthy alcohol use, Smoking

## Abstract

**Background:**

Visual analog scales (VAS) are sometimes used to assess change constructs that are often considered critical for change. Aims of Study: 1.) To determine the association of readiness to change, importance of changing and confidence in ability to change alcohol and tobacco use at baseline with the risk for drinking (more than 21 drinks per week/6 drinks or more on a single occasion more than once per month) and smoking (one or more cigarettes per day) six months later. 2.) To determine the association of readiness, importance and confidence with alcohol (number of drinks/week, number of binge drinking episodes/month) and tobacco (number of cigarettes/day) use at six months.

**Methods:**

This is a secondary analysis of data from a multi-substance brief intervention randomized trial. A sample of 461 Swiss young men was analyzed as a prospective cohort. Participants were assessed at baseline and six months later on alcohol and tobacco use, and at baseline on readiness to change, importance of changing and confidence in ability to change constructs, using visual analog scales ranging from 1–10 for drinking and smoking behaviors. Regression models controlling for receipt of brief intervention were employed for each change construct. The lowest level (1–4) of each scale was the reference group that was compared to the medium (5–7) and high (8–10) levels.

**Results:**

Among the 377 subjects reporting unhealthy alcohol use at baseline, mean (SD) readiness, importance and confidence to change drinking scores were 3.9 (3.0), 2.7 (2.2) and 7.2 (3.0), respectively. At follow-up, 108 (29%) reported no unhealthy alcohol use. Readiness was not associated with being risk-free at follow-up, but high importance (OR 2.94; 1.15, 7.50) and high confidence (OR 2.88; 1.46, 5.68) were. Among the 255 smokers at baseline, mean readiness, importance and confidence to change smoking scores were 4.6 (2.6), 5.3 (2.6) and 5.9 (2.7), respectively. At follow-up, 13% (33) reported no longer smoking. Neither readiness nor importance was associated with being a non-smoker, whereas high confidence (OR 3.29; 1.12, 9.62) was.

**Conclusions:**

High confidence in ability to change was associated with favorable outcomes for both drinking and smoking, whereas high importance was associated only with a favorable drinking outcome. This study points to the value of confidence as an important predictor of successful change for both drinking and smoking, and shows the value of importance in predicting successful changes in alcohol use.

**Trial registration number:**

ISRCTN78822107

## Background

Unhealthy alcohol use and smoking and its consequences represent a major burden of disease in the general population [1-3]. The consequences of heavy episodic drinking are of primary concern among young adults [4], and smoking is detrimental and affects the future health of young individuals [5-7]. To alleviate the impact on health behavior, counseling and brief interventions have been developed and have demonstrated evidence of efficacy in reducing alcohol use [8,9], including among young individuals [10,11]. For smoking, brief counseling interventions among young individuals and adolescents are promising (especially with the increasing use of electronic media tools), but evidence is very limited [12-15].

Within motivational intervention paradigms, clinicians are encouraged to assess client motivation toward changing unhealthy behaviors [16]. One’s readiness to change, importance of changing and confidence in ability to change are some of the various dimensions of substance use behaviors that have been explored [17-23]. Shifts in these dimensions are often considered intermediate goals on the way to achieving decreases in consumption [18,24,25]. In addition to being useful facilitators during clinical encounters, readiness, importance and confidence may have predictive value for future behavior change. Nevertheless, the focus on these dimensions and the use of readiness, importance and confidence as intermediate goals depends in large part on the assumption that there is a clear association between these dimensions and outcome. In fact, there is conflicting evidence about the predictive value of these dimensions. In addition, the value of a Transtheoretical Model (TTM) of change described by Prochaska and DiClemente [26] has been questioned [27-29]. Despite these challenges, various measures of readiness, importance and confidence are used in both clinical and research settings. Two recent studies conducted among adolescents attending treatment facilities showed the predictive validity of a readiness ruler on alcohol and tobacco use 6 months later [22,30]. The association between the readiness ruler and subsequent behavior change persisted at 12 months for alcohol, but not for tobacco. The predictive validity of a measure of confidence in ability to change tobacco use (10-point scale) 6 and 12 months later was also demonstrated.

In a population with unhealthy alcohol use, Korcha and colleagues found no association between readiness to change (measured with a 1–10 visual analog scale) and drinking (average number of drinks per drinking day and average number of drinking days per week) at 3 and 12 month follow-ups, but there was an association between readiness and maximum number of drinks on one occasion [31]. Similarly, Collins and colleagues did not show an association between readiness (measured with a readiness to change questionnaire) and drinking among students. Some additional studies suggest that these dimensions may operate differently in various populations and that some may play a more prominent role as predictors of future change than others [19,20].

A fuller understanding of the predictive power of those dimensions that impact young adults may help clinicians design more effective interventions, selecting those dimensions that are of primary interest and importance. Assuming that these dimensions do have predictive ability, they could be used to distinguish individuals likely to change with minimal intervention from those in need of additional support. Studying these dimensions across a number of different substances could also furnish clues to the intrinsic value of these dimensions vis-à-vis universal cognitive dimensions of behavior change. Therefore, we studied the readiness to change, importance of changing and confidence in ability to change constructs associated with alcohol and tobacco use. In a prospective cohort of 20-year-old men, we investigated the associations of these dimensions with drinking and smoking six months later. The aims of the present study were to determine the association of readiness to change, importance of changing and confidence in ability to change alcohol and tobacco use with the presence or absence of unhealthy alcohol use and smoking at follow-up, as well as the association of readiness, importance and confidence with continuous measures of alcohol use (number of drinks per week, number of binge drinking episodes per month) and tobacco use (number of cigarettes per day) at six months.

## Results

Twelve of the 853 potential subjects from the randomized controlled trial were dropped, due to missing values on baseline alcohol measures. Of the remaining 841, 577 reported unhealthy alcohol use and/or smoking at baseline, and 461 (80%) of them completed the six-month follow-up protocol and were included in the present study. Subjects who were not followed up did not differ from those who were with respect to baseline alcohol use (mean number of drinks per week, mean number of binge drinking episodes per month), smoking (mean number of cigarettes smoked per smoking day), or behavior change (readiness, importance, confidence) constructs (p > 0.10 for all measures). There were 206 subjects with unhealthy alcohol use only, 84 with smoking only and 171 with both. The baseline characteristics of these 461 participants are presented in Table 1.

**Table 1:** Baseline characteristics of subjects with unhealthy alcohol use and smoking (n = 461)

	Subjects with unhealthy alcohol use* (n = 206)	Subjects with smoking** (n = 84)	**Subjects with both risks (n = 171)**
Age, mean (SD)	20.0 (1.1)	20.4 (1.3)	20.0 (1.2)
Number of drinks per week, mean (SD)	13.8 (16.5)	3.7 (3.5)	14.9 (14.0)
Number of binge drinking episodes per month, mean (SD)	4.4 (3.4)	0.6 (0.5)	5.2 (4.5)
Number of cigarettes per days, mean (SD)	1.5 (3.4)	12.5 (6.3)	13.5 (7.3)
Education level, obligatory school only, n (%)	82 (39.8)	36 (42.9)	82 (48.0)
Occupation:			
In training, n (%)	150 (72.8)	57 (67.9)	117 (68.4)
Employed, n (%)	44 (21.4)	19 (22.6)	40 (23.4)
Inactive, n (%)	12 (5.8)	8 (9.5)	14 (8.2)
Readiness (alcohol), % low/medium/high	61/20/19		68/19/13
Importance (alcohol),% low/medium/high	83/12/5		82/13/5
Confidence (alcohol), % low/medium/high	16/18/66		26/23/51
Readiness (tobacco), % low/medium/high		51/33/16	52/31/17
Importance (tobacco), % low/medium/high		42/35/23	40/37/23
Confidence (tobacco), % low/medium/high		22/45/33	35/35/30

Note:

*: Unhealthy alcohol use was defined as drinking more than 21drinks per week or drinking 6 drinks or more on a single occasion more often than once per month.

**: Smoking was defined as smoking at least one cigarette per day.

For readiness, importance and confidence: low (1–4), medium (5–7), high (8–10), recoded from 1–10.

### Subjects with unhealthy alcohol use at baseline

Among the 377 subjects reporting unhealthy alcohol use at baseline, mean (SD) readiness, importance and confidence to change drinking scores were 3.9 (3.0), 2.7 (2.2) and 7.2 (3.0), respectively. At six months, 108 (29%) reported no unhealthy alcohol use.

Primary outcome: Table 2 presents the results of the logistic regressions. In separate models adjusting for receipt of brief intervention, there was no association between readiness and being risk-free for alcohol use six months later. Subjects with high importance (OR 2.94; 1.15, 7.50) and high confidence (OR 2.88; 1.46, 5.68) levels were more likely to be risk-free compared to subjects with low importance and low confidence levels. Given the number of subjects with and without unhealthy alcohol use at follow-up, and since the three change dimensions were not highly correlated (i.e. Spearman coefficients for readiness-importance: 0.37, readiness-confidence: 0.21 and importance-confidence: 0.13), a model including all three dimensions and adjusted for receipt of brief intervention and smoking status at baseline was computed. The results (not shown in Table 2) were similar to the three models computed separately and adjusted for receipt of BI and smoking status at baseline, indicating an independent association of high importance and high confidence with favorable alcohol outcomes at follow-up: the adjusted OR (95%CI) for a favorable drinking outcome (i.e. no longer reporting unhealthy alcohol use) at follow up were 1.08 (0.57, 2.06) and 0.89 (0.45, 1.76) for medium and high readiness (low = reference group), 0.95 (0.43, 2.11) and 3.09 (1.10, 8.68) for medium and high importance (low = reference group), 2.11 (0.91, 4.91) and 2.91 (1.44, 5.85) for medium and high confidence (low = reference group).

**Table 2:** Association between readiness, importance and confidence and favorable outcomes at six months

	**Subjects with unhealthy alcohol use**	**Subjects with smoking**
	Separate logistic regression models (one model for each construct, each model was adjusted for receipt of BI and smoking risk status at baseline), AOR (95%CI)*	Separate logistic regression models (one model for each construct, each model was adjusted for receipt of BI and alcohol risk status at baseline), AOR (95%CI)*
Readiness (reference group: low)	Model 1	Model 1
Medium	1.26 (0.71, 2.22)	2.05 (0.89, 4.70)
High	1.42 (0.78, 2.58)	2.07 (0.76, 5.68)
Importance (reference group: low)	Model 2	Model 2
Medium	0.91 (0.45, 1.84)	1.41 (0.58, 3.43)
High	2.94 (1.15, 7.50)	2.10 (0.83, 5.29)
Confidence (reference group: low)	Model 3	Model 3
Medium	2.16 (0.97, 4.78)	2.18 (0.74, 6.45)
High	2.88 (1.46, 5.68)	3.29 (1.12, 9.62)

* All models were adjusted for receipt of a brief intervention and smoking risk status at baseline (for subjects with unhealthy alcohol use) and unhealthy alcohol use (for subjects with smoking). Reporting no unhealthy alcohol use/smoking less than 1 cigarette a day (favorable outcome) was coded 1 in the logistic regression model.

Unhealthy alcohol use was defined as drinking more than 21 drinks per week or drinking 6 drinks or more on a single occasion more often than once per month. Smoking was defined as smoking at least one cigarette per day.

For readiness, importance and confidence: low (1–4), medium (5–7), high (8–10), recoded from 1–10.

Secondary outcomes: Table 3 shows that readiness and importance were not associated with number of drinks per week or number of binge drinking episodes per month at follow-up. Compared to subjects with low confidence levels, those with high confidence levels at baseline reported 20-30% fewer drinks per week and binge episodes per month, and these associations were significant.

**Table 3:** Association between readiness, importance and confidence and drinking and smoking at six months

**Readiness, importance, and confidence to change drinking**	**Number of drinks/week**		**Number of binge drinking episodes/month**	
	IRR (95%CI)	p	IRR (95%CI)	p
Readiness (reference group: low)				
Medium	0.92 (0.74, 1.13)	0.40	0.99 (0.78, 1.24)	0.91
High	1.07 (0.86, 1.34)	0.53	1.08 (0.85, 1.38)	0.52
	IRR (95%CI)	p	IRR (95%CI)	p
Importance (reference group: low)				
Medium	1.11 (0.87, 1.42)	0.40	1.11 (0.85, 1.45)	0.43
High	1.00 (0.70, 1.45)	0.98	1.07 (0.71, 1.60)	0.75
	IRR (95%CI)	p	IRR (95%CI)	p
Confidence (reference group: low)				
Medium	0.84 (0.66, 1.08)	0.18	0.82 (0.62, 1.06)	0.13
High	0.80 (0.65, 0.98)	0.03	0.74 (0.59, 0.92)	0.008

Readiness, importance, and confidence to change smoking	Number of cigarettes/smoking day			
	IRR (95%CI)	p	IRR (95%CI)	p
Readiness (reference group: low)				
Medium	1.03 (0.88, 1.20)	0.73		
High	0.98 (0.81, 1.18)	0.79		
	IRR (95%CI)	p	IRR (95%CI)	p
Importance (reference group: low)				
Medium	0.99 (0.85, 1.15)	0.92		
High	1.08 (0.91, 1.28)	0.36		
	IRR (95%CI)	p	IRR (95%CI)	p
Confidence (reference group: low)				
Medium	0.96 (0.82, 1.13)	0.62		
High	0.91 (0.77, 1.08)	0.29		

IRR: incidence rate ratio.

All models were negative binomial regression models. Analyses conducted for subjects with unhealthy alcohol use were adjusted for receipt of brief intervention, drinking at baseline (number of drinks per day or number of binge drinking episodes per month) and smoking status at baseline. Analyses conducted for subjects with smoking were adjusted for receipt of brief intervention, smoking at baseline (number of cigarettes per smoking day), and presence of unhealthy alcohol use at baseline.

For readiness, importance and confidence: low (1–4), medium (5–7), high (8–10), recoded from 1–10.

### Subjects smoking at baseline

Among the 255 smokers at baseline, mean readiness, importance and confidence to change smoking scores were 4.6 (2.6), 5.3 (2.6) and 5.9 (2.7), respectively. At six months, 33 (13%) reported no longer smoking.

Primary outcome: Table 2 presents the results of the logistic regressions. Neither readiness nor importance was associated with being a non-smoker, whereas high confidence was (OR 3.29; 1.12, 9.62). Given the number of non-smokers at follow-up, it was not possible to include all three of the behavior change measures in a single regression model.

Secondary outcome: Table 3 shows that there were no significant associations of readiness, importance, or confidence with number of cigarettes smoked per smoking day at follow-up.

## Discussion

We investigated the association of three behavior change constructs (readiness, importance and confidence) with drinking and smoking behaviors. In this prospective cohort sample, it appears that changes in alcohol use are far more frequent than changes in smoking; while 29% of the subjects with baseline unhealthy alcohol use were no longer drinking unhealthy amounts at follow-up, only 13% of the baseline smokers no longer smoked at least one cigarette per day.

These results demonstrate that high confidence levels were associated with subsequent changes in drinking and smoking risk status. The magnitudes of association were similar for both behaviors, i.e. subjects who had high confidence in their ability to change were about three times more likely to no longer report the target behavior (unhealthy alcohol use, daily smoking) than were subjects with low confidence levels. Confidence appears to be a stable predictor of subsequent reductions in both alcohol and tobacco use. These results were confirmed in secondary analyses of alcohol use; subjects with high confidence levels reported fewer drinks per week and fewer binge episodes per month than did subjects with low confidence levels. Results also suggest that there is a dose–response relationship between confidence and drinking outcomes. For smoking, results of the primary outcome were not confirmed in the secondary analyses. Nevertheless, the measure of effect suggests a dose–response relationship between confidence and number of cigarettes smoked per smoking day, even if the association failed to reach statistical significance.

Our findings are consistent with other reports that point out the potential role of confidence in ability to change as a good predictor of subsequent change [20,22]. They can be linked to other studies conducted in other populations (such as adults or females) that show the impact of self-efficacy on relapse and abstinence for both smoking and drinking [32-35].

The importance of changing results were mixed. For primary outcomes, there was an association of high importance with favorable changes in drinking, but not with smoking. There were no associations found for secondary outcomes.

Readiness to change did not seem to be associated with changes in either drinking or smoking, and is seemingly at odds with current behavior change theories. Past research among adolescents has shown an association between readiness to change (measured with a ruler) and actual changes in alcohol and tobacco use [22,30]. Unlike those in our sample, the Maisto and Chung subjects were recruited at substance use treatment facilities, so it is possible that the research setting influenced the results. In addition, the authors used a slightly different ruler than was used herein. It should be noted that the confidence scale in the Chung study is comparable to ours, and yielded similar results. The psychometric properties of the readiness rulers may be influenced by differences in wording, e.g. “10” in the Maisto study corresponds to “trying hard to change”, whereas “10” in ours corresponds to “ready to change”. However, other studies also failed to show an association between readiness to change and behavior change [20,31,36,37]. As opposed to confidence, readiness may reflect severity of use rather than a dimension associated with the ability to enact changes [19,38], but there is no definitive answer with respect to the predictive value of readiness. Heterogeneity in the measures across various studies is a likely explanation. Similarly, readiness may operate differently within different populations, e.g. treatment/non-treatment or specialized treatment milieus, etc.). In addition, most of the research conducted on readiness to change refers to the Transtheoretical Model (TTM), whose value has been questioned [27]. However, our study was not intended to be a validation of TTM. Even though measures we used relate more or less to some of the processes described in TTM, we did not assess the experiential and behavioral components comprising TTM. We evaluated whether clinical measures were associated with subsequent substance use, so our results yield useful information for clinical and predictive utility. Since the component constructs of motivation to change (readiness, importance, and confidence) are not highly correlated and predict independently in a combined regression model, they could be measured and used separately in research and practice. A consistent finding of ours across the two substances studied is that individuals with higher levels of confidence were more likely to modify their substance use than were those reporting lower levels of confidence; this may help identify individuals who need more support in order to change their alcohol and tobacco use. Our results question the utility of relying on readiness to change as a predictor of substance use.

There are several limitations to consider in this study. First, our subjects agreed to participate in research allowing them to receive a brief motivational intervention, and thus might have been predisposed to changing. In addition, secondary analyses of randomized trial data can present methodological challenges, and sample size was determined for the randomized trial, not for the present analyses (note: at alpha level 0.05, the power to detect differences observed for the main outcomes of unhealthy alcohol use and smoking was 100%). However, unlike secondary analyses in other cohort designs, the intervention is well-specified and its recipients are in an identified group where all of the analyses can be controlled for intervention delivery. The fact that all analyses were adjusted for the receipt of brief intervention and they showed only a small, non-significant intervention effect [39], makes it unlikely that our results are the consequence of receiving a brief intervention. Furthermore, efforts were made in this randomized trial within this particular population to ensure an acceptable follow-up rate of 80%. Nevertheless, the sample was small, and the study needs replication using larger samples. An additional limitation is the use of self-reported measures of alcohol and tobacco use. There is a long-standing debate about the reliability of self-reports of substance use [40-43]. Despite its limitations, self-report remains an affordable, acceptable and often used method of assessing substance use. Self-report may be subject to social desirability and recall biases (especially for substances like alcohol that may affect memory); consequently, substance use may be under-reported, especially in situations involving social pressure or disapproval [44]. In the present study, efforts were made to assure confidentiality, and a non-judgmental approach to discussing substance use was stressed in order to limit the risk of bias, but we cannot rule out the potential for inaccurate reporting among participants.

Since our study included only young men, our results should not be seen as generalizable to women or older adults, whose drinking and smoking habits may differ and for whom readiness, importance and confidence may play a different role. Generalizability is limited to young men agreeing to discuss their substance use, since subjects who agree to participate in a study where they have to talk about their substance use are likely to differ on motivation and substance use from those who do not. Even though recruited at a site where virtually all Swiss young men could be contacted, those willingly participating differed slightly from the total population. As reported for the randomized controlled trial, 22.1% of those available for participation were interested in receiving a brief motivational intervention. Since 98.6% of non-participants in that trial completed a short screening questionnaire, we were able to compare them to participants, who had a higher prevalence of smoking (54.4% vs. 47.7%). The prevalence of drinking >21 drinks per week was similar (9.6%) between the two groups, but the prevalence of binge drinking was higher (55.3 vs. 49.8%) among participants compared to non-participants [39].

Our study also has several noteworthy strengths. We used a non-clinical sample of young men at the Lausanne army recruitment center that processes all French-speaking Swiss males in order to assess eligibility for military service. This procedure is mandatory in Switzerland and is a unique opportunity to contact a population-based sample. Typically, individuals in this setting do not seek treatment and seldom access primary care services. Other population-based studies evaluating behavior change constructs are relatively scarce.

## Conclusions

Whether there is a causal relationship between confidence in ability to change and subsequent changes in drinking and smoking, or whether changes in confidence can lead to greatly improved outcomes remains to be seen. Nevertheless, this report adds to the body of evidence suggesting that confidence and self-efficacy are critical dimensions that may be causally linked to behavior change. Although assessing readiness and the importance of changing may have some clinical utility, determining confidence in ability to change may be a better predictor of future improvements in alcohol and tobacco use, and may relate more specifically to individual capacity for successful change.

## Methods

The present study is a secondary analysis of data from a randomized trial [39]. The sample was analyzed as a prospective cohort; eligible subjects for the present study were included regardless of the randomization group in the intervention trial. Subjects were drawn from a large prospective cohort of 20-year-old Swiss men attending the army recruitment center in Lausanne, Switzerland, who participated in a randomized controlled trial of the impact of a multi-substance brief motivational intervention. Among 8,419 potentially eligible subjects, 3,652 were not available due to logistical constraints. Of the remaining 4,767, 1,052 (22.1%) were interested in receiving a brief motivational intervention and 853 were ultimately included in the trial [39].The randomized trial tested the intervention’s primary and secondary effects of preventing increases in substance use for those without unhealthy use, and decreasing substance use for those who with unhealthy use, thus all individuals were included regardless of their usage. All subjects gave written informed consent, and the Ethics Committee for Clinical Research at the Lausanne University Medical School approved the study. The army was blinded to the data collection during the research. Within the larger study, subjects were randomized to receive either a brief motivational intervention or not, and participation was not restricted to those smoked or were unhealthy alcohol users, whereas in the present study only those who smoked or had unhealthy alcohol use were selected. Subjects were eligible if they reported drinking more than 21 drinks per week, or had more than one episode of six or more drinks (one drink = 10g of ethanol) per occasion per month, or smoked one or more cigarettes per day. Before being assigned to a group, all subjects completed a baseline assessment that included demography (age, occupation, and education level), measures of alcohol and tobacco use, and behavior change items. All subjects were intended to be followed up in six months.

### Measures

Subjects were assessed on each of the three behavior change constructs using visual analog scales ranging from 1 ("not ready/not important to change/not confident to succeed") to 10 ("ready/very important to change/very confident to succeed") for smoking and for alcohol use (total of 6 scales). The questions were: "how ready are you to change your drinking/smoking habits"; "how important is it for you right now to change your drinking/smoking"; and "if you decide to change your drinking/smoking habits, how confident are you that you would succeed”. Answers were later recoded into three categories: low (1–4), medium (5–7) and high (8–10). This procedure has been used by Korcha and colleagues [31] and has the advantage of giving clinicians a directly useful measure versus a categorization based on the distribution of the variables, such as tertiles or quartiles. Although allowing comparability between dimensions, analyses by groups based on distributions were not used; categories that have potential clinical significance were chosen instead.

Evaluating change dimensions is contingent on individuals presenting with unhealthy behaviors. Therefore, readiness, importance and confidence responses were retained only for those who met our definition of unhealthy behaviors for drinking and smoking (i.e. more than 21 drinks per week, or more than one episode of six or more drinks per occasion per month, and smoking one or more cigarettes per day).

### Outcomes

"First research question: what is the association between readiness to change, importance of changing and confidence in ability to change alcohol and tobacco use and the presence of unhealthy alcohol use and smoking 6 months later?"

In order to compare the predictive value of the three behavior change constructs across the two substance use behaviors, a dichotomous risk status outcome was calculated for both smoking and alcohol use. At six months, subjects were classified as having unhealthy alcohol use if they reported drinking more than 21 drinks per week or having more than one episode with six or more drinks per occasion per month (e.g. binge drinking). They were classified as smokers if they reported smoking at least one cigarette per day.

"Second research question: what is the association between readiness, importance and confidence and continuous measures of alcohol use (number of drinks per week, number of binge drinking episodes per month) and tobacco use (number of cigarettes per day) 6 months later?"

The secondary outcomes were the number of drinks per week, the number of binge drinking episodes per month and the number of cigarettes smoked per smoking day.

### Statistical analyses

Analyses were conducted separately for subjects with unhealthy alcohol use and for those who smoked daily.

Primary outcomes: Logistic regressions were used to assess the relationship between each behavior change construct and subsequent unhealthy substance use at the six-month follow-up. All of the models were adjusted for the receipt of brief intervention. In addition, analyses conducted on subjects with unhealthy alcohol use were adjusted for the presence of smoking risk status, while analyses for subjects who smoked were adjusted for the presence of unhealthy alcohol use. When an adequate number of subjects existed and a correlation coefficient >0.4 between dimensions was absent, a model containing all three constructs was computed, then adjusted for receipt of a brief intervention and smoking/alcohol risk status at baseline. The combined regression was then compared to the separate logistic models for each of the three change dimensions. Due to the small number of non-smoking subjects at six months, we did not run the model containing all three constructs among smokers.

Secondary outcomes: the number of drinks per week, the number of binge drinking episodes per month and the number of cigarettes smoked per smoking day were analyzed using negative binomial regression models (NBRM) to assess their relationship to each behavior change construct. Comparisons using standard test BIC/AIC (Bayesian and Akaike information criteria) showed that NBRM was a better fit than Poisson regression (PRM), zero-inflated Poisson (ZIP) or zero-inflated negative binomial (ZINB) models. Analyses conducted for subjects with unhealthy alcohol use were adjusted for receipt of brief intervention, drinking at baseline and smoking status at baseline. Analyses conducted for subjects with smoking were adjusted for receipt of brief intervention, baseline number of cigarettes per smoking day, and presence of unhealthy alcohol use at baseline.

SAS software 9.2 (Cary, North Carolina) was used for these analyses, and P values less than 0.05 were considered to be statistically significant.

## Competing interests

The authors declare that they have no conflict of interest.

## Role of funding source

The study was funded by the Tobacco Prevention Fund from the Swiss Government (REF 240.0007-4/08.001190/510491). The Tobacco Prevention Fund had no influence on the research and results presented in this paper.

## Authors’ contributions

NB: conception and design of the study, acquisition, analysis and interpretation of data, main drafting of the manuscript; JG: acquisition and interpretation of data, drafting and revision of the manuscript; MF: design of the study, analysis and interpretation of the data, drafting and revision of the manuscript; GG: conception of the study, acquisition and interpretation of data, revision of the manuscript; JBD: conception of the study, acquisition and interpretation of data, revision of the manuscript. All authors read and approved the current version of the manuscript.

## Authors information

Submitted for publication to BMC Public Health, February 2012.

## Pre-publication history

The pre-publication history for this paper can be accessed here:

http://www.biomedcentral.com/1471-2458/12/708/prepub
